# Every breath you don’t take, I’ll be helping you: Ethylene promotes hypoxia tolerance

**DOI:** 10.1093/plphys/kiac347

**Published:** 2022-07-27

**Authors:** José Manuel Ugalde

**Affiliations:** INRES—Chemical Signalling, University of Bonn, Bonn 53113, Germany

After drought, flooding is the gravest natural disaster that endangers agriculture and food security worldwide. Losses due to flooding reached USD 21 billion between 2008 and 2018, representing 19% of all total agricultural losses in low and lower middle-income countries (FAO, 2021). Upon flooding, plants encounter restricted gas diffusion, leading to oxygen (O_2_) deprivation, passing from 21% (normoxia) to <1% oxygen availability when submerged (hypoxia). To keep cells alive under hypoxic conditions, plants must metabolically switch from mitochondrial respiration and carbon fixation to glycolysis and ethanolic fermentation for energy production (Sasidharan et al., 2018).

During submergence, plants use dynamic variations in the levels of O_2_, reactive oxygen species (ROS), nitric oxide (NO), and the gaseous phytohormone ethylene as intracellular cues to adjust to flooding conditions. Impeded gas diffusion in water promotes entrapment of ethylene in the plant, leading to increases of up to 20-fold in concentration within the first hour of submergence compared to nonsubmerged tissues (Sasidharan et al., 2018). In the model plant Arabidopsis (*Arabidopsis thaliana*), pretreatments with ethylene enhanced hypoxia tolerance, increasing rosette sizes and the capacity of the root tip to re-grow during recovery, henceforth root tip survival (Hartman et al., 2019). However, the mechanisms behind ethylene-mediated hypoxia survival are still unclear.

In this issue of *Plant Physiology*, Liu et al. (2022) studied the mechanisms by which ethylene pretreatments help Arabidopsis root tip survival during hypoxia and re-oxygenation. Because re-oxygenation after hypoxia triggers a ROS burst that could damage the cell, the authors evaluated cell viability at different recovery timepoints after a 4-h hypoxia treatment. Cell death was observed as soon as 1 h into recovery, and cell death was alleviated in plants pretreated with ethylene, leading to enhanced cell survival. The authors then investigated which processes are associated with ethylene-induced tolerance to hypoxia and re-oxygenation. To address this question, they sampled the root tips of Arabidopsis seedlings pretreated with ethylene or air before and after hypoxia treatments and 1 h after re-oxygenation. They performed a comprehensive analysis of gene expression and proteome variations in the root tip in all these conditions.

Ethylene stabilizes group VII ethylene response factors (ERFVIIs) by inducing expression of PHYTOGLOBIN1 (PGB1), a NO-scavenger (Hartman et al., 2019). ERFVIIs are a component of the O_2_-/NO-sensing mechanism, and continuous proteasome degradation via PROTEOLYSIS6 and the N-degron pathway limits ERFVII accumulation (Licausi et al., 2011; Gibbs et al., 2014). Hence, submergence-induced ethylene accumulation in the cell promotes nuclear translocation of stabilized ERFVIIs transcription factors, upregulating the expression of hypoxia-response genes.

Most of the genes differentially expressed during hypoxia were already differentially expressed immediately after the ethylene pretreatment, indicating that ethylene signaling triggers a transcriptome reconfiguration maintained during hypoxia. Enriched gene ontology (GO) terms of genes upregulated by ethylene treatments were linked to hypoxia response and abscisic acid (ABA), among others. In comparison, GO terms enriched in the downregulated genes were related to decreased cellular maintenance and root growth, such as *PLETHORA* (*PLT*) *1* and *2*, *SCARECROW* (*SCR*), and *SHORTROOT* (*SHR*). Moreover, ethylene limited the expression of genes related to ROS homeostasis and antioxidant activity, such as peroxidases like *ASCORBATE PEROXIDASE 2* (*APX2*) (Figure 1).

**Figure 1:**
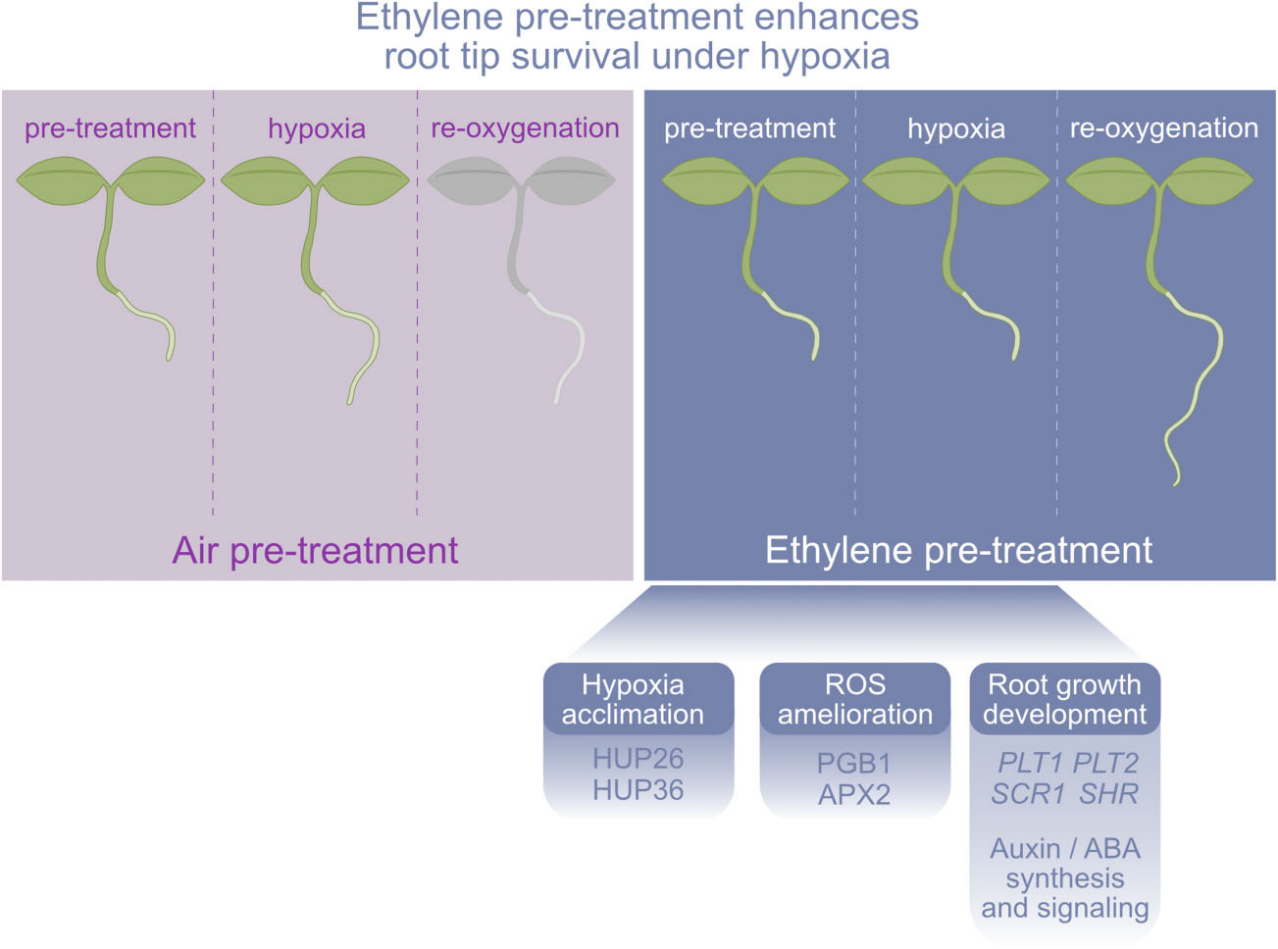
Ethylene modulates various responses to enhance plant tolerance to hypoxia and re-oxygenation. Ethylene accumulation promotes hypoxia tolerance via several strategies: enhanced proteins levels for components of the core hypoxia acclimation response (PGB1, HUP26, and HUP36); managing ROS homeostasis by inducing the accumulation of the NO-scavenger, PGB1, and many peroxidases, like APX2; and the restriction of root growth by modulating the expression of genes related to root development (*PLT1*, *PLT2*, *SCR1*, and *SHR*) while controlling the biosynthesis and signaling of hormones such as ABA and auxin. Image modified from Liu et al. (2022).

Translation is restricted during severe hypoxia (Sachs et al., 1980), yet de novo synthesis of a set of proteins is essential for plant acclimation and survival under low oxygen conditions. Therefore, the authors focused on identifying how the proteome of root tips changes after pretreatment with ethylene using tandem mass tag labeling and quantitative mass spectrometry. Proteins with increased abundance were linked to processes such as protein folding and anaerobic metabolism, while abundance of proteins linked to DNA replication and translation was decreased. Among the top most differentially enriched proteins after ethylene pretreatment were PGB1, HYPOXIA-RESPONSIVE UNKNOWN PROTEIN 26 (HUP26), and HUP36 (Figure 1), which are core hypoxia-responsive genes.

Since ethylene alters the expression and protein abundance of several peroxidases, the authors explored the role of ethylene in ROS homeostasis during hypoxia and re-oxygenation. Ethylene pretreatments increased the total peroxidase activity in root tips while increasing the levels of glutathione, yet not changing the redox state of the glutathione pool. Furthermore, by using the mitochondria-targeted biosensor, roGFP2-Orp1 (Nietzel et al., 2019), the authors found that ethylene pretreatments abolished hypoxia-related ROS production in the mitochondria. The authors showed that ethylene-mediated decreases in ROS production are not restricted to hypoxia-induced ROS only. Challenging root tips with externally applied H_2_O_2_ demonstrated that ethylene pretreatments increased root tip survival after H_2_O_2_ treatments. Furthermore, they showed that the ethylene-mediated protective effect depends on the cell’s ability to sense the hormone via its receptor and trigger a downstream signaling pathway that includes the NO-scavenger PGB1 and the enhanced stability of ERFVIIs such as RELATED TO APETALA 2.2 (RAP2.2) and RAP2.12.

Finally, the authors tested how ethylene-mediated root growth inhibition functions as an additional strategy through which ethylene pretreatments save plant resources and energy during hypoxia. Ethylene pretreatments decreased root tip growth, improving their hypoxia survival (Figure 1). This growth inhibition was lesser in an *AUXIN INFLUX CARRIER PROTEIN 1* null allele, *aux1-22*, which is deficient in the upregulation of auxin-responsive genes (Swarup et al., 2007) but responds to the ethylene-induced changes.

Taken together, Liu et al. (2022) provided detailed evidence on how ethylene pretreatments are sufficient to reprogram root cells, helping the plant survive during and after hypoxia. The hormone promotes cellular processes linked to the central hypoxia response and restricts other operations related to cell maintenance and root growth. Such a strategy places ethylene as a critical trigger of targeted signaling in the onset of hypoxia, optimizing resources in an energetically challenging scenario. Using comprehensive transcriptomic and proteomic analyses allows us to begin understanding ethylene-induced genes for which functions remain unknown. Also, genetically encoded sensors targeting different sub compartments, such as the endoplasmic reticulum or the cytosol, can provide further information on spatial ROS dynamics during hypoxia and re-oxygenation and how ethylene alleviates the production of these ROS upon hypoxia.

*Conflict of interest statement.* None declared.

## References

[kiac347-B1] FAO (2021) The Impact of Disasters and Crises on Agriculture and Food Security: 2021. FAO Rome, Italy

[kiac347-B2] Gibbs DJ , Md Isa N, Movahedi M, Lozano-Juste J, Mendiondo GM, Berckhan S, Marín-de la Rosa N, Vicente Conde J, Sousa Correia C, Pearce SP, et al (2014) Nitric oxide sensing in plants is mediated by proteolytic control of group VII ERF transcription factors. Mol Cell 53: 369–379 24462115 10.1016/j.molcel.2013.12.020 PMC3969242

[kiac347-B3] Hartman S , Liu Z, van Veen H, Vicente J, Reinen E, Martopawiro S, Zhang H, van Dongen N, Bosman F, Bassel GW, et al (2019) Ethylene-mediated nitric oxide depletion pre-adapts plants to hypoxia stress. Nat Commun 10: 4020 31488841 10.1038/s41467-019-12045-4 PMC6728379

[kiac347-B4] Licausi F , Kosmacz M, Weits DA, Giuntoli B, Giorgi FM, Voesenek LACJ, Perata P, van Dongen JT (2011) Oxygen sensing in plants is mediated by an N-end rule pathway for protein destabilization. Nature 479: 419–422 22020282 10.1038/nature10536

[kiac347-B5] Liu Z , Hartman S, van Veen H, Zhang H, Leeggangers HACF, Martopawiro S, Bosman F, de Deugd F, Su P, Hummel M, et al (2022) Ethylene augments root hypoxia tolerance through amelioration of reactive oxygen species and growth cessation. Plant Physiol 190: 1365--1383 10.1093/plphys/kiac245 PMC9516759 35640551

[kiac347-B6] Nietzel T , Elsässer M, Ruberti C, Steinbeck J, Ugalde JM, Fuchs P, Wagner S, Ostermann L, Moseler A, Lemke P, et al (2019) The fluorescent protein sensor roGFP2-Orp1 monitors *in vivo* H_2_O_2_ and thiol redox integration and elucidates intracellular H_2_O_2_ dynamics during elicitor-induced oxidative burst in Arabidopsis. New Phytol 221: 1649–1664 30347449 10.1111/nph.15550

[kiac347-B7] Sachs MM , Freeling M, Okimoto R (1980) The anaerobic proteins of maize. Cell 20: 761–767 7418006 10.1016/0092-8674(80)90322-0

[kiac347-B8] Sasidharan R , Hartman S, Liu Z, Martopawiro S, Sajeev N, van Veen H, Yeung E, Voesenek LACJ (2018) Signal dynamics and interactions during flooding stress. Plant Physiol 176: 1106–1117 29097391 10.1104/pp.17.01232 PMC5813540

[kiac347-B9] Swarup R , Perry P, Hagenbeek D, Van Der Straeten D, Beemster GTS, Sandberg G, Bhalerao R, Ljung K, Bennett MJ (2007) Ethylene upregulates auxin biosynthesis in Arabidopsis seedlings to enhance inhibition of root cell elongation. Plant Cell 19: 2186–2196 17630275 10.1105/tpc.107.052100 PMC1955695

